# Arming Inactivated Enveloped Virus Vaccines with the *GGTA1* Gene: A Potent Method for Amplification of Viral Vaccines Effectiveness and Protection Against Variants

**DOI:** 10.3390/vaccines14070571

**Published:** 2026-06-29

**Authors:** Uri Galili

**Affiliations:** Rush University Medical Center, Chicago, IL 60612, USA; uri.galili@rcn.com

**Keywords:** anti-Gal, α-gal epitope, α1,3galactosyltransferase, COVID-19, *GGTA1* gene, SARS-CoV-2 virus, Ebola virus, vaccine immunogenicity, variants

## Abstract

This review describes a novel method for increasing the effectiveness of inactivated enveloped whole-virus vaccines by targeting them for extensive uptake by antigen-presenting cells (APCs). Several inactivated whole-virus vaccines with dense glycan shields display suboptimal effectiveness because the multiple carbohydrate chains (glycans) on the virus mask immunogenic peptides and surround the virus with a negative electrostatic charge that decreases uptake by APCs. It is postulated that engineering such vaccinating viruses to present the carbohydrate antigen “α-gal epitope” on the glycan shields will immunocomplex them with the anti-Gal antibody; thus, it will target them for robust uptake by APCs. Anti-Gal is an abundant natural antibody in humans, constituting ~1% of human circulating immunoglobulins. The ligand of anti-Gal is the α-gal epitope, which is naturally synthesized in non-primate mammals and New World monkeys by the glycosylation enzyme α1,3galactosyltransferase. This enzyme is encoded by the *GGTA1*-gene. Viral vaccines presenting multiple α-gal epitopes on their glycan shield bind anti-Gal and activate the complement system to produce complement chemotactic cleavage peptides C5a and C3a that induce extensive recruitment of APCs to vaccine injection sites. The virion-bound anti-Gal further targets the viral vaccine for robust uptake by APCs, following binding of its Fc “tail” to Fcγ-receptors on APCs. The efficacy of this method was studied in anti-Gal-producing mice with α-gal presenting inactivated influenza virus vaccine and with gp120 of HIV presenting this epitope. These studies indicated that virus vaccines engineered to present α-gal epitopes increase anti-virus antibody production and virus-specific T-cell activation by 15- to 100-fold in comparison to the same vaccines lacking α-gal epitopes. It is suggested that α-gal presenting inactivated SARS-CoV-2 virus vaccines can induce a similar protective long-term immune memory against S- M-, E-, and N-viral proteins. Furthermore, immune-escaping variants of the mutated S-protein may be destroyed by antibodies to M and E proteins, and cells infected with such variants may be killed by cytotoxic T cells specific to peptides of the N-protein. Such an anti-M-, E-, and N-protein immune protection may prevent expansion of these variants and thus may avoid the need for immunization with COVID-19 vaccines every 6 months or following the appearance of new variants. A similar potent immunization may be achieved with an inactivated *Ebolavirus* vaccine engineered to present α-gal epitopes on the glycan shield. The resulting immune response to the various *Ebolavirus* proteins also may contribute to cross-reactive protection against other *Ebolavirus* species containing proteins with evolutionarily conserved structures. An effective method for the preparation of a whole-virus vaccine presenting α-gal epitopes is by arming it with the *GGTA1*-gene inserted into the viral genome. Such virions will present multiple α-gal epitopes on their glycan shield, which will amplify their immunogenicity instead of reducing it in the wild-type virus.

## 1. Introduction

Viral vaccines may include live-attenuated viruses, inactivated (killed) viruses, parts of the virus (e.g., envelope glycoproteins), DNA, or mRNA of a major envelope glycoprotein. Some of the inactivated whole-virus vaccines have been reported to be only of suboptimal effectiveness. Such is the example of COVID-19 SARS-CoV-2 inactivated virus vaccines, which were found in phase III randomized clinical trials to display in different countries protective efficacy of 50–90% [1,2,3,4,5,6]. Thus, improving the effectiveness of such vaccines, in medium- and low-income countries, is needed [1]. The main approach for increasing the effectiveness of viral vaccines is by increasing their immunogenicity. It is well recognized that one of the main causes for decreased immunogenicity of enveloped virus vaccines is the glycan shield on the virus envelope [7,8,9,10,11]. The glycan shield is formed by the many carbohydrate chains (glycans) that are linked to the viral envelope proteins. This review describes studies that have led to the development of a novel type of viral vaccines (called α-gal viral vaccines) by engineering their glycan shields to present the carbohydrate antigen called “α-gal epitope”. The studies described below, with α-gal epitopes on influenza virus vaccine [12] and on gp120 of HIV vaccine [13], indicated that their immunogenicity increased by 15–100-fold in comparison to similar vaccines lacking α-gal epitopes. In view of these studies, it is suggested that arming vaccinating enveloped viruses with the *GGTA1* gene, which encodes the enzyme synthesizing α-gal epitopes (α1,3galactosyltransferase [α1,3GT]), converts the glycan shield of α-gal viral vaccines from a masking component of the vaccine into a component that greatly increases vaccine immunogenicity by robust targeting of inactivated virions to antigen-presenting cells (APCs). This review further hypothesizes that such α-gal SARS-CoV-2 and α-gal Ebola virus vaccines may elicit potent immune responses with long-term immune memory against conserved viral antigens, thereby protecting against future SARS-CoV-2 variants and cross-protecting against Ebola virus species not included in vaccines.

## 2. The Glycan Shield Protects Enveloped Viruses

**Figure 1:**
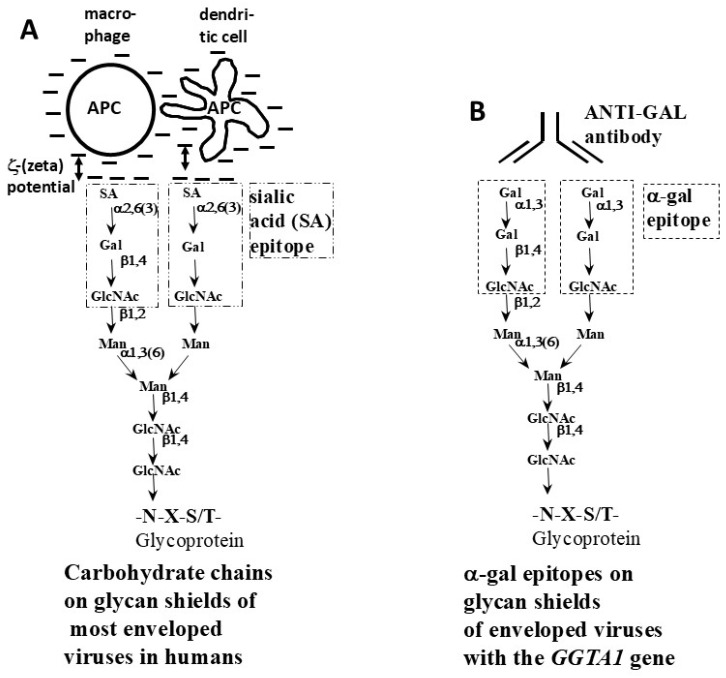
N-linked glycans of the complex type presented on enveloped virus glycoproteins and discussed in this review. (**A**) Glycan capped with sialic acid (SA). It is suggested that the glycan shield containing these glycans decreases the immunogenicity of vaccinating inactivated enveloped viruses, which are deflected from APCs by the ζ (zeta) potential between the negative electrostatic charges of SA on the viral envelope and those of SA on APC membranes. It is further suggested that this deflection decreases the number of vaccinating virions taken up by pinocytosis into the APCs. (**B**) Glycan shields presenting α-gal epitopes bind the natural anti-Gal antibody. This antibody opsonizes the vaccinating virions, thereby mediating extensive binding of the virions to Fcγ receptors on APCs, which results in robust uptake of these virions by the APCs.

Many enveloped viruses decorate their envelope proteins with multiple glycans. The viral glycans are synthesized within the Golgi apparatus of the host cell by cellular enzymes called glycosyltransferases. These enzymes add carbohydrate units to the nascent glycan on cellular or viral glycoproteins in a sequential manner, like an assembly line in a car plant. Many of these glycans are N (asparagine)-linked, and their number per glycoprotein molecule varies from one type of virus to another. For example, there are 5–7 N-linked glycans on hemagglutinin of influenza virus [14,15,16], 22 on S-protein of SARS-CoV-2 [7,17], 24 on gp120 of HIV [18,19], and 17 on the envelope glycoprotein of Ebola virus [20,21]. The asparagine amino acids that serve as a glycosylation site for glycans on viral envelopes are those of the sequon Asn-X-Ser/Thr (i.e., asparagine–any amino acid–serine or threonine). One of the most common envelope N-glycans on viruses is the glycan of the “complex type” that is capped by terminal negatively charged sialic acid (also called N-acetyl neuraminic acid). The sialic acid is presented on glycans with two, three or four antennae of N-linked glycans (a glycan with two antennae capped with sialic acid is illustrated in Figure 1A). The enveloped glycoproteins on some viruses, which are considered to be among the most dangerous, are densely glycosylated with such sialic acid-capped glycans, including HIV, SARS-CoV-2 and Ebola virus [7,17,18,19,20,21].

Because the glycans are hydrophilic, they protrude from the envelope proteins like quills of a porcupine, and thus, they can sterically mask host immune recognition of protein antigens on the viral envelope. This results in low immunogenicity of the virus, and it contributes to virus escape from neutralizing antibodies [1,7,8,9,10,11,22]. This low immunogenicity is further assisted by the fact that the viral glycans are non-immunogenic because they are the same as those on the host cells, and thus it helps the virus to persist longer in infected hosts without significant detection by the immune system. This contribution of the glycan shield to viral immune evasion can be observed in the viral evolution of HIV and Influenza A virus into more virulent viruses by mutations that add N-glycan sites to gp120 of HIV [10] and to hemagglutinin of influenza virus [15].

The glycan shield also decreases the immunogenicity (i.e., effectiveness) of inactivated whole enveloped virus vaccines. Vaccinating virions must be internalized by APCs at the vaccination site, where they are administered by injection. Internalized virions are transported by the APCs to regional lymph nodes. These APCs process viral peptides and present them on cell surface MHC molecules. The presented peptides activate CD4^+^ and CD8^+^ T-cells. CD8^+^ T-cells with T cell receptors (TCR) specific to the viral antigens are activated to proliferate and mature into cytotoxic T cells (CTL). Upon subsequent infection of the vaccinated individuals by the corresponding live virus, these CTL detect virus-infected cells and kill them, thereby preventing further expansion of the infecting virus. The activated CD4^+^ T cells become helper T cells that secrete cytokines, which assist virus-specific CD8^+^ T cell clones to expand by proliferation and become memory CTL that are activated upon infection by the virus. The CD4^+^ helper T cells also help activate virus-specific B cells for their maturation into plasma cells producing anti-virus neutralizing antibodies and into memory B cells.

An effective viral vaccine requires extensive uptake of the immunizing virions or viral protein by APCs. The glycan shield affects the uptake efficacy of vaccinating virions by APCs via the following mechanisms: 1. Masking of the vaccinating virion antigens, and 2. Electrostatic repulsion by sialic acid.

### 2.1. Masking Viral Antigens

As indicated above, a large proportion of the surface of the main envelope glycoprotein in some viruses is covered by a dense glycan shield that masks viral antigenic peptides [7,8,9,10,11]. These glycans are identical to the self-glycans on the various cells in humans since they are synthesized by the glycosylation machinery of the host cell. In the absence of opsonizing markers that identify the vaccinating virions, their uptake by APCs is mediated only by random pinocytosis, i.e., internalization of virions that happen to be very close to the APC membrane. This random mechanism results in the uptake by APCs of a relatively small proportion of the vaccinating virions. An increased number of APCs at the vaccination site and active targeting of the vaccinating virions to APCs can increase vaccine effectiveness.

### 2.2. Electrostatic Repulsion by Sialic Acid

As shown in Figure 1A, many of the N-linked glycans on viral envelope glycoproteins infecting human cells are capped by the negatively charged carbohydrate sialic acid (SA). In addition, SA is also found capping a large proportion of N-glycans on the surface of cells of the various tissues, including APCs and red blood cells. The negative electrostatic charges on red blood cells and on endothelial cells cause mutual deflection due to the repulsion between the same electrostatic charges (called ζ [zeta]-potential). When the SA presentation is enzymatically decreased on the surface of red blood cells [23] or decreased by infecting *Plasmodium* parasite [24], the red blood cells display abnormal adhesion to endothelial cells, caused by the lack of sufficient repulsion due to a lack of ζ potential. It is hypothesized that the ζ potential between SA on enveloped viruses and APCs decreases the immunogenicity of viral vaccines. Multiple SA units are presented on glycan shields as a result of the synthesis of viral glycans by the glycosyltransferases in the host cell Golgi apparatus. As indicated above, APCs internalize vaccinating viruses by random pinocytosis of virions, which accidentally get very close to the APC cell membrane. However, when vaccinating virions, surrounded by the multiple negative charges of SA on their glycan shield, get close to the APC cell membrane, the mutual negative electrostatic charges of SA on the virions and on APCs are likely to cause deflection of these virions from the APCs by the ζ potential, like the same poles of two magnets deflect each other. It is suggested that this physical phenomenon decreases the overall number of vaccinating virions internalized by APCs, thereby decreasing the vaccinating efficacy of inactivated whole-virus vaccines with dense glycan shield.

The present review describes a novel method for the conversion of the glycan shield on vaccinating virions into a part of the vaccine presenting α-gal epitopes (illustrated in Figure 1B). These epitopes bind the natural anti-Gal antibody, thereby inducing both extensive recruitment of APCs into the vaccination site and robust targeting of such vaccinating virions for their active uptake by the recruited APCs.

## 3. The Natural Anti-Gal Antibody and the α-Gal Epitope

Anti-Gal is one of the most abundant natural antibodies in humans, constituting as much as ~1% of circulating immunoglobulins in all humans who are not severely immunocompromised, regardless of blood type [25,26,27,28]. Anti-Gal is produced throughout life in response to antigenic stimulation by carbohydrate antigens on human microbiota [29,30,31,32,33,34]. Anti-Gal is also produced in Old World monkeys (monkeys of Asia and Africa), and apes (gibbons, orangutans, gorillas and chimpanzees) [35,36]. The carbohydrate antigen (ligand) that binds the natural anti-Gal antibody is the tri-saccharide Galα1-3Galβ1-4GlcNAc, called the α-gal epitope (Figure 1B) [37,38]. This tri-saccharide is naturally found on glycoproteins, glycolipids and proteoglycans of part of mammals, but is absent in other vertebrates. Among mammals, the α-gal epitope is synthesized in non-primate mammals, lemurs, and New World monkeys, but is absent in Old World monkeys, apes and humans [35,39,40].

The α-gal epitope is synthesized by the glycosylation enzyme α1,3galactosyltransferase (α1,3GT) [39,41,42,43,44] in the following reaction:

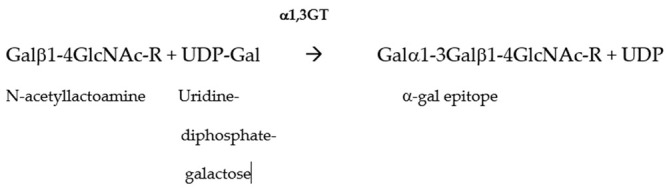

α1,3GT is encoded by the *GGTA1* gene [45,46]. *GGTA1* is found to be an inactivated gene in humans, apes and Old World monkeys [47,48,49,50]. The inactivation of this gene due to base deletions seems to have occurred 20–30 million years ago and was immediately followed by the production of the natural anti-Gal antibody in mutated ancestral Old World monkey and ape offspring (Old World primates) [51].

Since the glycans on enveloped viruses are synthesized by the glycosylation machinery of host cells, viruses that infect any non-primate mammal present α-gal epitopes on a proportion of their glycans, in addition to SA and other carbohydrate epitopes. α-Gal epitopes on viral envelope glycoproteins were reported on viruses propagated in cells containing active α1,3GT, including Friend murine leukemia virus propagated in mouse cells [52] and influenza virus produced in bovine and canine cells [53]. Furthermore, any virus that replicated in non-primate mammalian cells or in human cells transfected with α1,3GT cDNA was also found to present α-gal epitopes and thus to bind the anti-Gal antibody within human serum [54,55,56,57,58,59,60,61]. These viruses were neutralized by complement activation when incubated in human serum at 37 °C. The activated complement cascade bores “holes” in the envelope of viruses, resulting in their destruction.

Domesticated animals, including cattle, horses, sheep, goats, and pigs, as well as pets such as dogs and cats, all have active α1,3GT. Thus, it is likely that viruses infecting these mammals express α-gal epitopes on their envelope. In view of the virolytic effect of anti-Gal in human serum that was described above, it was suggested that this antibody contributes to the protection against infections by zoonotic viruses replicating in non-primate mammals [60]. In addition, it was suggested that anti-Gal appeared as a natural antibody in a few ancestral mutated Old World primates in which the *GGTA1* gene was accidentally inactivated, thus, they lost the α-gal epitope [51]. This antibody protected such mutated primates from infection by lethal viruses, which killed the non-mutated parental hosts that synthesized α-gal epitopes and thus lacked the protective anti-Gal antibody. As a result of these evolutionary endemic events in the landmass of Europe, Asia and Africa, parental Old World primate populations were eliminated and replaced by anti-Gal-producing primates because their *GGTA1* gene was inactivated.

## 4. Hypothesis on Anti-Gal-Mediated Amplification of Inactivated Virus Vaccine Effectiveness

**Figure 2:**
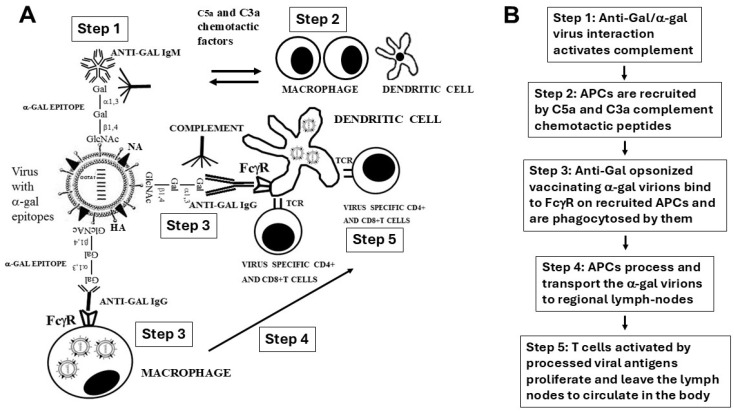
Hypothesis on the increased immunogenicity of α-gal virus vaccines. (**A**) Illustration of the steps in the mechanism that increases the number of APCs recruited to the vaccination site and the robust targeting of the vaccinating virions to APCs, using α-gal virions as a vaccine. The influenza virus is used as a representative virus for illustrating the proposed mechanism. (**B**) A flow-chart of the various steps leading to increased immunogenicity of the α-gal virus vaccine. HA—hemagglutinin, NA—neuraminidase, FcγR—Fcγ receptor, TCR—T-cell receptor. Modified from the book by Galili U: *The Natural Anti-Gal Antibody as Foe Turned Friend in Medicine*, Academic Press/Elsevier, 2018, with permission.

As discussed above, the immunogenicity of inactivated vaccinating enveloped virions can be greatly amplified if the number of APCs is increased at the vaccination site and vaccinating virions are actively targeted for robust uptake by APCs. Both objectives can be achieved by using two basic immunological principles: 1. Antigen/antibody interaction activates the complement system, and the newly formed C5a and C3a complement cleavage peptides can serve as potent chemotactic factors that recruit APCs to the complement activation site. 2. Vaccinating virions opsonized by an IgG antibody are effectively targeted for uptake by APCs via Fc/Fcγ receptors (FcγR) interaction. The proposed hypothesis postulates that insertion of the *GGTA1* gene into the genome of vaccinating viruses will result in presentation of α-gal epitopes on a proportion of the envelope’s glycans. These epitopes will induce conversion of the immunogenicity-reducing glycan shield into a component of the vaccinating virus that induces both extensive recruitment of APCs into the vaccination site and robust uptake of anti-Gal-opsonized virions by the recruited APCs. This extensive uptake of many virions by APCs is postulated to result in activation and proliferation of many T and B cells specific to most or all the multi-antigens in the vaccinating virus. It is further postulated that such an immune response will induce an effective long-term immune memory for protection against subsequent viral infections. Such an immune response will result in rapid production of high-affinity antibodies to the virus envelope proteins and in the appearance within a short time of CTL that kill virus-infected cells presenting peptides of envelope, nucleocapsid and other internal proteins of the virus, on their MHC. This hypothesis is illustrated in Figure 2A, in which an inactivated influenza virus presenting α-gal epitopes serves as a representative vaccinating virus, referred to as an α-gal virus, and in Figure 2B, which presents a flow chart of this hypothesis.

In Step 1 of Figure 2A, the anti-Gal antibody binds to α-gal epitopes on vaccinating virions. This interaction activates the local complement system. In Step 2, chemotactic complement cleavage peptides C5a and C3a, which are generated as part of the activated complement cascade, direct the extensive recruitment of the APCs dendritic cells and macrophages to the vaccinating α-gal virions. In Step 3, the Fc “tail” of anti-Gal IgG-coating vaccinating α-gal virions interacts with the Fcγ receptor (FcγR) on recruited APCs, resulting in phagocytosis of the virions by these APCs. This step loads the APCs with a much higher amount of internalized vaccinating α-gal virions than with virions lacking α-gal epitopes and thus, not opsonized by anti-Gal. Binding of the C3b complement molecule on the vaccinating virus to complement receptor 1 (CR1) on APCs also contributes to uptake of the vaccinating virions. In Step 4, the APCs transport internalized virions to the regional lymph nodes, process viral antigenic peptides, and present them on class I and class II HLA molecules. In Step 5, processed viral antigenic peptides, presented on class I HLA molecules, activate the virus-specific CD8^+^ T-cells to proliferate and mature as CTL killing virus-infected cells. Viral antigenic peptides presented on class II HLA molecules activate virus-specific CD4^+^ T cells, which proliferate and secrete cytokines that help B-cell and T-cell responses. Overall, it is postulated that α-gal virus vaccines mediate the mechanism in Figure 2A for achieving a much higher effectiveness in the induction of protective anti-virus humoral and cellular immune responses with longer immune memory than the same vaccines lacking α-gal epitopes.

The increased effectiveness of α-gal virus vaccines could be demonstrated with anti-Gal-producing mice [62] in the sections below. The postulated extensive recruitment of APCs by α-gal virions and their targeting for uptake by APCs could be demonstrated using α-gal nanoparticles comprised of phospholipids, cholesterol, and α-gal glycolipids presenting multiple α-gal epitopes on their surface [63]. These α-gal nanoparticles simulate inactivated α-gal viruses, in that they present α-gal epitopes and have a size of 100–300 nm. The experimental animals used for these studies were mice in which the *GGTA1* gene was inactivated, thus they lack α-gal epitopes and are called GT-KO mice [62]. These mice produce anti-Gal in titers similar to those in humans, following immunization with pig kidney membranes homogenate presenting multiple α-gal epitopes [63].

**Figure 3:**
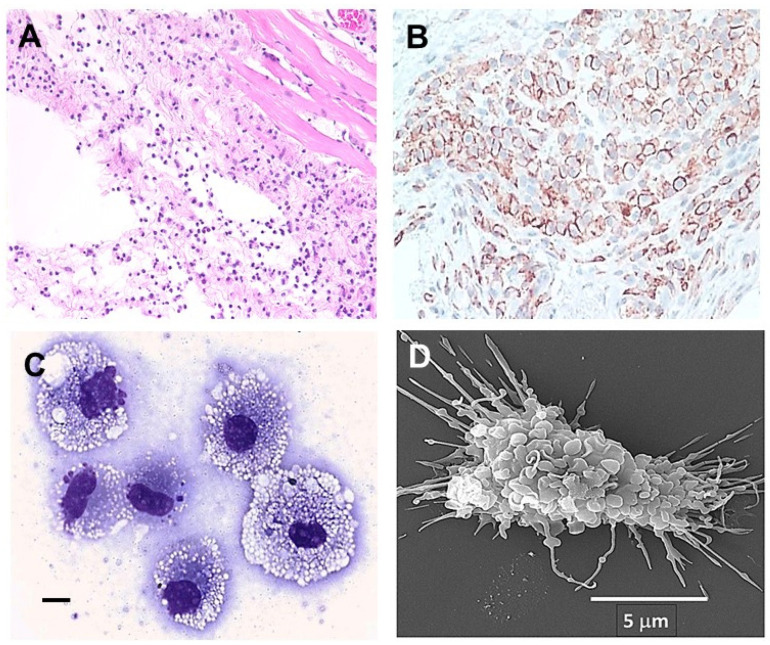
Simulation of anti-Gal/α-gal virus vaccine interaction mediating recruitment of macrophages and Fc/FcγR binding to macrophages, using α-gal nanoparticles. (**A**) Recruitment of macrophage (most cells in areas with no fibroblasts), 24 h after intradermal injection into GT-KO mice of 1.0 mg α-gal nanoparticles suspension in saline. The empty area was filled with α-gal nanoparticles that were dissolved by alcohol in the fixation stage of staining (hematoxylin & eosin [H&E] ×100). (**B**) Macrophage infiltration on day 4. Cells were immunostained with the macrophage-specific anti-F4/80 antibody (peroxidase immunostaining ×400). (**C**) Recruited macrophages after 7 days. The macrophages are filled with vacuoles due to the uptake of anti-Gal-opsonized α-gal nanoparticles. The bar represents 10 μm. (Giemza ×1000). (**D**) Scanning electron microscopy of a representative macrophage covered with anti-Gal-opsonized α-gal nanoparticles (small spheres) because of anti-Gal/FcγR interactions. Adapted from the book by Galili U: *The Natural Anti-Gal Antibody as Foe Turned Friend in Medicine*, Academic Press/Elsevier, 2018, with permission.

Intradermal injection of α-gal nanoparticles into anti-Gal-producing GT-KO mice results in extensive recruitment of macrophages within 24 h, by the anti-Gal/α-gal nanoparticle interaction (Figure 3A). This activity simulates a similar anti-Gal-mediated recruitment of α-gal virions illustrated in Step 2 of Figure 2A. Such recruitment increases after 4 days (Figure 3B), and most of the recruited cells are stained by the macrophage-specific anti-F4/80 antibody. The number of recruited macrophages peaks after a week, and their higher magnification at that time point reveals large, activated macrophages with multiple vacuoles, which represent the many phagocytosed anti-Gal-opsonized α-gal nanoparticles (Figure 3C).

Simulation of Step 3 in Figure 2A is shown in Figure 3D, in which anti-Gal-coated α-gal nanoparticles were incubated with adherent macrophages for 2 h. After additional extensive washes, the macrophages were processed for analysis by scanning electron microscopy. The α-gal nanoparticles are the small spheres covering the surface of the representative macrophage. They adhere to the macrophage following binding of the Fc tail of the anti-Gal coating α-gal nanoparticles to FcγR on macrophages. In the absence of anti-Gal, no nanoparticle adhesion to the macrophages is observed [64]. The same Fc/FcγR interaction between the α-gal virions and the recruited APCs results in the extensive uptake of the vaccinating viral antigens by the APCs (Figure 3C), and thus, much larger amounts of antigens are transported to the regional lymph nodes. This robust uptake results in a more potent immune response induced by the vaccine, in comparison to viral vaccines lacking α-gal epitopes [12,13].

Marked increases in immunogenicity of antibody opsonized vaccinating antigens due to Fc/FcγR binding to APCs were observed with a variety of immunocomplexed antigens [65,66,67,68,69,70]. The unique advantage of the anti-Gal-mediated targeting of the vaccine to APCs, in comparison to the other studies on antibody-mediated vaccine targeting to APCs, is that in humans, anti-Gal is already present in all individuals undergoing vaccination and does not require any immunologic manipulation, other than the use of α-gal-presenting vaccines.

## 5. Anti-Gal-Mediated Increased Transport of Immunizing Antigens to Lymph Nodes

Step 4 in Figure 2A postulates that the robust uptake of vaccinating α-gal viruses by APCs is followed by transport of increased amounts of the viral antigens to the regional lymph nodes. This step could be demonstrated with chicken ovalbumin (OVA) as an immunizing antigen encapsulated within small liposomes presenting α-gal epitopes (OVA-liposomes), which simulate α-gal virus vaccines [71]. OVA processed and presented by APCs can be detected by presentation of the peptide SIINFEKL on MHC. This presented peptide activates CD8^+^ T cells with TCR specifically binding SIINFEKL presented on MHC. APCs presenting SIINFEKL are experimentally detected by activation of the T hybridoma cell line B3Z to proliferate, since the TCR specific for SIINFEKL is presented by these hybridoma cells [72,73]. Anti-Gal-producing GT-KO mice were immunized with OVA-liposomes presenting α-gal epitopes. B3Z cells were co-incubated with cells from vaccine-draining lymph nodes of the immunized mice. This co-incubation indicated that the number of SIINFEKL-presenting APCs in these mice was 7–8-fold higher than in immunized mice lacking the anti-Gal antibody [71]. Accordingly, the number of vaccine-specific T cells evaluated by ELISPOT was higher by 15-fold, and anti-OVA antibody production was higher by 30–100-fold, in comparison to corresponding results in mice lacking anti-Gal, following vaccination with OVA-liposomes [71]. Thus, the observed anti-Gal-mediated targeting of immunizing OVA-containing α-gal-liposomes to APCs implies that anti-Gal is a key component in the amplification of α-gal vaccine effectiveness.

## 6. Amplification of α-Gal Influenza Virus Vaccine Immunogenicity

Step 5 in Figure 2A could be evaluated by measuring the increase in the immune response following immunization with an α-gal virus vaccine in comparison to the same vaccine that lacks α-gal epitopes. This was evaluated with a vaccine prepared from a heat-inactivated influenza virus strain A/Puerto Rico/8/34-H1N1 (PR8 virus). The inactivated vaccinating virions were engineered to present α-gal epitopes (referred to as PR8_α-gal_) by recombinant (r)α1,3GT and UDP-Gal, as indicated in the reaction above [12].

**Figure 4:**
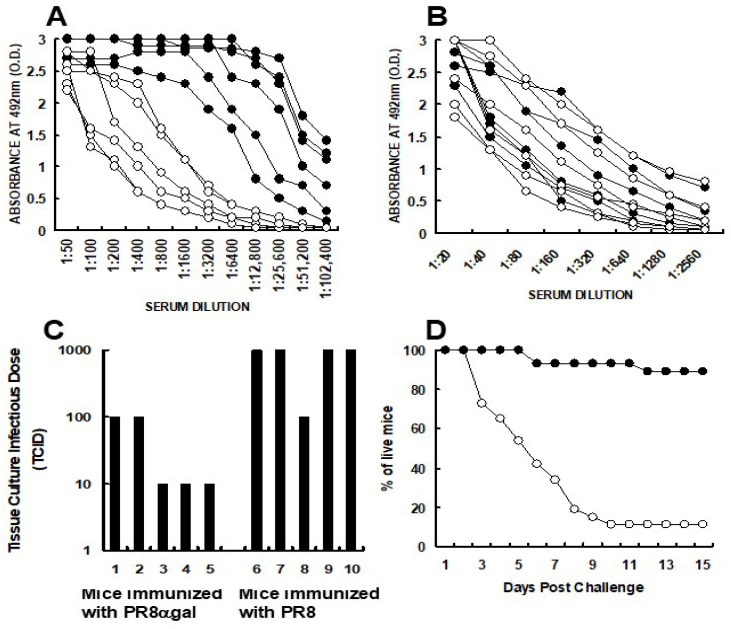
Increased immunogenicity of PR8_α-gal_ inactivated whole-virus vaccine. (**A**) Anti-PR8 influenza virus IgG antibodies produced in anti-Gal-producing GT-KO mice immunized twice with inactivated PR8_α-gal_ vaccine (●) (*n* = 6) or with inactivated PR8 vaccine (○) (*n* = 6 per group). Antibodies were assayed by ELISA with PR8 virus as the solid-phase antigen. (**B**) Anti-PR8 antibody production in wild-type mice immunized as in (**A**). Note that in the absence of anti-Gal, there is no difference in antibody production between the two groups. (**C**) Proliferation of PR8 virus in the lungs of GT-KO mice vaccinated with PR8_α-gal_ or with PR8 inactivated virus vaccines, 3 days post challenge with a lethal dose of PR8 virus and evaluated as reciprocal of cytopathic tissue culture infection dose (TCID) in MDCK cell cultures (*n* = 5 per group). (**D**) Survival of mice immunized twice with inactivated PR8 (○) or with inactivated PR8_α-gal_ (●) and challenged with a lethal dose of live PR8 virus. (*n* = 25/group). Data presented as % of mice surviving the challenge at different time points. Survival did not alter post day 15. Adapted from ref. [12] with permission.

GT-KO mice producing anti-Gal were immunized twice with 1 μg PR8_α-gal_ vaccine in a two-week interval, or with 1 μg PR8 vaccine lacking α-gal epitopes. The vaccines also included Ribi (trehalose dicorynomycolate) adjuvant. Anti-PR8 antibody production was assessed in the sera two weeks following the second immunization, by ELISA with PR8 virus as the solid phase antigen. As shown in Figure 4A, the activity of the anti-virus antibody in PR8_α-gal_-immunized mice was ~100-fold higher than that in mice immunized with the PR8 virus lacking α-gal epitopes [12]. Similar immunizations in wild-type mice resulted in low anti-PR8 antibody production in mice immunized with PR8_α-gal_ as well as in those immunized with PR8 (Figure 4B). This implied that in the absence of anti-Gal, no amplification of the immune response is observed with vaccinating virions presenting α-gal epitopes.

The differences in the immune response to PR8_α-gal_ vs. that to PR8 vaccines in anti-Gal-producing GT-KO mice were further reflected in the progression of live PR8 challenge infection. The immunized mice were challenged intranasally with a lethal dose of live PR8 virus, two weeks after the second immunization. Three days later, the lungs were harvested, and the amount of live PR8 virus was measured in the minced lungs. Live viruses were quantified by virus cytopathic tissue culture infection dose (TCID) in MDCK cell monolayers. The number of infectious PR8 virions retrieved from the lungs of PR8-immunized mice was 10- to 100-fold higher than that of infectious PR8 virions retrieved from the lungs of PR8_α-gal_-immunized GT-KO mice (Figure 4C). Immunized mice were also challenged intranasally with a lethal dose of infectious PR8 virus (i.e., dose killing 100% of non-immunized GT-KO mice) and monitored for survival. As many as of 89% of PR8-immunized mice were killed by the challenging virus within 15 days, whereas only 11% of the PR8_α-gal_-immunized mice were killed by this challenge (Figure 4D) [12].

The studies with the PR8 virus indicate that an inactivated α-gal virus vaccine induces a much higher protective immune response against subsequent infections with live virus than an inactivated virus vaccine lacking α-gal epitopes. This increased immune response is dependent on immune complexing of the α-gal virus vaccine with anti-Gal. It is of note that capsid proteins of non-enveloped viruses (e.g., adenovirus or poliovirus) usually lack glycans. Thus, covalent attachment of α-gal epitopes to amino acids of capsid proteins may change their antigenicity so that the produced antibodies and the activated T cells may be directed toward the antigens changed by the covalent linker rather than toward the non-engineered viral antigens. However, no changes in protein antigenicity occur when the glycan shield is engineered by rα1,3GT to present α-gal epitopes since there are no alterations in the amino acids structure.

## 7. Amplification of HIV α-Gal gp120 Vaccine Immunogenicity

The principle of increased immunogenicity of α-gal vaccines is applicable not only to enveloped viruses, but also to individual glycoprotein molecule vaccines. This was demonstrated with recombinant gp120, the envelope glycoprotein of HIV. This glycoprotein has 24 N-linked glycans, at least half of which are capped with sialic acid (SA) units (Figure 1A) [18,19]. The SA on gp120 could be replaced by α-gal epitopes (gp120_α-gal_) using a combination of neuraminidase and rα1,3GT [13]. Subcutaneous immunization of anti-Gal-producing GT-KO mice was performed with 5 μg gp120 or gp120_α-gal_ in Ribi adjuvant and was repeated after two weeks. The number of gp120-specific T cells and anti-gp120 antibody production were evaluated 17 days following the second injection.

**Figure 5:**
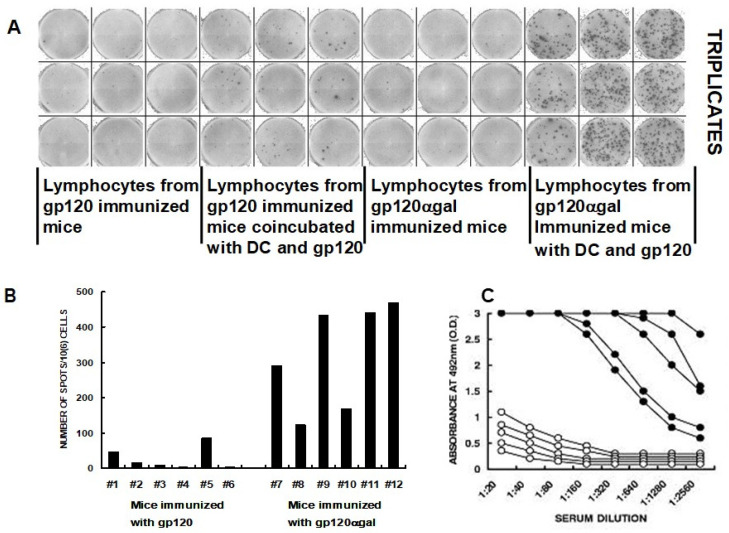
Increased immunogenicity of gp120_α-gal_ vaccine. (**A**) ELISPOT analysis in triplicate wells (vertical lanes), of IFN-γ secretion by activated gp120-specific T cells in the spleens of mice immunized twice with 5 μg gp120 or gp120_α-gal_. Analysis was performed in the presence or absence of gp120-pulsed dendritic cells (DC). Each dot represents one activated T cell secreting IFN-γ. (**B**) Summary of ELISPOT data for 6 mice immunized with gp120 (mice 1 to 6) or gp120_α-gal_ (mice 7 to 12), as the number of spots per 10^6^ splenocytes. (**C**) Anti-gp120 antibody production in GT-KO mice following two immunizations with gp120 or gp120_α-gal_. Each dot represents one activated T cell secreting IFN-γ. Adapted from ref. [13] with permission.

T-cell response to the gp120 vaccines was determined in ELISPOT assays by quantification of IFN-γ-secreting cells among spleen cells of the immunized GT-KO mice co-incubated with gp120-pulsed dendritic cells. Whereas the number of IFN-γ-secreting T cells in gp120 immunized mice was on average 23 per 10^6^ splenocytes, in gp120_α-gal_-immunized mice, it was 332 per 10^6^ cells (Figure 5A,B) [13]. Accordingly, the titer of anti-gp120 IgG antibodies, as measured by ELISA with gp120 as the solid-phase antigen, was on average ~100-fold higher in gp120_α-gal_-immunized mice than in gp120-immunized mice. Both anti-gp120 antibody and ELISPOT data demonstrate extensive amplification of humoral and cellular immune responses against viral antigens if the vaccinating antigens are engineered to present α-gal epitopes and thus are effectively targeted to APCs by the anti-Gal antibody.

The sera in gp120_α-gal_-immunized mice neutralized HIV at titers of 200–600, whereas sera of the gp120-immunized group displayed neutralizing HIV at titers of only ≤10 [13]. Overall, the studies on gp120_α-gal_ vaccine suggest that anti-Gal-mediated increased effectiveness of vaccines can be achieved with both intact viruses and soluble viral proteins engineered to present α-gal epitopes.

## 8. The α-Gal Epitope as a Universal Augmenter of Vaccine Immunogenicity

The principle of increasing vaccine immunogenicity by converting it to an α-gal vaccine was found to be applicable in different kinds of vaccines, in addition to viral vaccines. An example of any soluble protein vaccine is bovine serum albumin (BSA). This protein was reported to display a marked increase in its immunogenicity in anti-Gal-producing GT-KO mice if it presents glycans with α-gal epitopes that were covalently linked to it [74]. Tumor antigens in cancer vaccines engineered to present α-gal epitopes were also found to be converted into effective anti-tumor vaccines. Engineering tumor cells to present α-gal epitope was found to induce a protective immune response against tumor antigens in these vaccinating cells, without having to characterize these antigens in mice [63,74,75,76,77,78] and in humans, by a mechanism similar to that illustrated in Figure 2 [79,80,81,82,83]. Moreover, since the α-gal epitope was found to be presented on protozoa such as *Plasmodium*, *Trypanosoma*, and *Leishmania*, it has been suggested that vaccines containing α-gal epitopes can elevate anti-Gal titers, thereby inducing prophylactic protection against such protozoa [34,84,85,86,87,88,89,90].

## 9. Inactivated COVID-19 α-Gal Whole-Virus Vaccines Preventing Appearance of Future Variants

The α-gal viral vaccine may be of particular significance in immunization of large populations for the prevention of future COVID-19 pandemics. mRNA vaccines of the S-protein were found to be effective and are used for protection against SARS-CoV-2 infections. However, this protective effectiveness (i.e., immune memory) usually decreases and requires a booster after ~6 months [1,2,3]. This need for repeated vaccinations is hard to follow in low-income countries and in poor communities [1]. The need for boosters every 6 months of mRNA vaccines may be associated with suboptimal APC uptake of spike (S)-proteins produced in the vaccinated individuals. Thus, the number of T and B cells activated by the vaccinating S-protein produced following the mRNA vaccine may be insufficient for maintaining a long-term immune memory. As discussed above, suboptimal immunogenicity of S-protein requiring 6-month booster, may be associated with the dense glycan shield masking immunogenic peptides and the electrostatic negative charge surrounding the S-protein and generated by ~22 SA residues on its glycans [17]. As illustrated in Figure 1A, the negative electrostatic charges of these many SA units may decrease the uptake of immunizing S-protein molecules by the ζ (zeta)-potential, which can deflect this protein from the surface of APCs. It is hypothesized that such electrostatic repulsion can reduce the number of protein molecules internalized into APCs by pinocytosis.

Mutations within the S-protein gene were found to generate several SARS-CoV-2 variants that are not sensitive to the protective effects of anti-S-protein antibodies produced following mRNA vaccination [91,92,93,94,95,96,97]. SARS-CoV-2 was found capable of tolerating multiple mutations without being inactivated. This is exemplified in the appearance of Omicron subvariant BA.1, displaying ~50 mutations in comparison to the wild-type (Wuhan) strain, of which 30 mutations in the S-protein were predicted to influence antibody neutralization and spike function [93,94,95,96,97]. This variant has ~10-times higher transmissibility than the wild-type strain, and it can infect non-vaccinated individuals and those vaccinated with currently used gene-based vaccines. This tolerance to mutations raises the possibility that poor populations infected by SARS-CoV-2 and living in areas with dust containing radionuclides in the air (e.g., originating from soil removed from mines) are prone to mutations of the virus that generate variants. This mutability can be caused by the small distance between infecting virions and radionuclides containing dust particles trapped in the mucous lining the airways and emitting mutagenizing radiation [98]. The repeated appearance of variants strongly suggests that virions containing escape mutations in the S-protein are a moving target that is selected for preferred expansion and transmission in mRNA-immunized populations, as well as in unimmunized populations [99,100]. It is reasonable to assume that the development of highly immunogenic multi-antigenic COVID-19 vaccines, comprised of S-protein and other SARS-CoV-2 antigens, may solve the problem of having to immunize populations with a new mRNA vaccine each time a new highly transmissible SARS-CoV-2 variant of concern appears in any part of the world.

The most likely multi-antigenic COVID-19 vaccine that comprises all SARS-CoV-2 antigens is the inactivated whole-virus vaccine. However, such vaccines displayed in phase III clinical trials variable efficacy outcomes—50.7% in Brazil [4], 65.3% in Indonesia [5], and 83.5% in Turkey [6]. Based on the studies above with α-gal influenza [12] and α-gal gp120 vaccines [13], it is hypothesized that whereas an inactivated SARS-CoV-2 whole-virus vaccine has suboptimal efficacy, a similar vaccine that presents multiple α-gal epitopes (referred to as SARS-CoV-2_α-gal_) may induce a protective immune response and long-term memory against the various proteins of the virus. It is further hypothesized that such a potent immune response against SARS-CoV-2 multi-antigens will also induce a long-term immune memory that enables protection against variants with mutations in the S-protein. This suggestion is supported by the observations that a number of childhood vaccines, such as measles, mumps, rubella and chicken-pox, are potent enough to induce a protective cellular and humoral immune memory for life. Additional supportive observations are the reports indicating that in convalescent patients of SARS-CoV-2 infections, the immune system produces antibodies to the envelope M- and E-proteins and displays elevated cytotoxic T-cell (CTL) response against the N-protein. This immune response is produced together with the immune response against the S (spike)-proteins [101,102,103,104,105]. Based on these observations and on the experience gained with PR8_α-gal_ and with gp120_α-gal_ described above, it is postulated that an inactivated whole SARS-CoV-2_α-gal_ vaccine is likely to be effective in eliciting a protective long-term immune memory against the various proteins of this virus. Since the M-, E- and N-proteins are conserved in SARS-CoV-2 [106,107,108,109], the probability that they will co-mutate in variants with S-protein is very low. Therefore, it is hypothesized that the anti- M-, E- and N-immune response in individuals immunized with the SARS-CoV-2_α-gal_ vaccine may be potent enough to neutralize future variants having immune-escape mutations in the S-protein.

In view of these considerations, it is hypothesized that use of the suggested SARS-CoV-2_α-gal_ vaccine may contribute to public health in various countries on three levels: 1. The potent immune response to the SARS-CoV-2_α-gal_ vaccine, due to the effective APC-mediated transport and processing of large amounts of the internalized vaccinating virions, will result in long-term effective immune memory that protects against recurrent infections by SARS-CoV-2. 2. The SARS-CoV-2_α-gal_ vaccine may serve as an effective multi-antigenic vaccine that, in addition to protection against the S-protein, may induce a protective immune response against the highly conserved M-, E- and N-proteins. This immune response may neutralize future variants containing immune-escape mutations in the S-protein, before they multiply and cause new epidemics. 3. In SARS-CoV-2_α-gal_ vaccinated individuals, the long-term immune memory against S-, M-, E- and N-proteins may negate the need for boosters every 6 months and for production of vaccines against each of the future COVID-19 variants. A method for engineering SARS-CoV-2 virions to present α-gal epitopes and serve as SARS-CoV-2_α-gal_ inactivated whole-virus vaccines is described below.

## 10. Suggested Arming Inactivated Whole-Virus Vaccine with the *GGTA1* Gene: SARS-CoV-2_α-gal_ as an Example

It is suggested that the most direct engineering of a vaccinating SARS-CoV-2 virus and other enveloped viruses for presenting α-gal epitopes is by arming the virus with the open reading frame of the *GGTA1* gene obtained from a non-primate mammal. It is postulated that insertion of the *GGTA1* gene into SARS-CoV-2 for the formation of SARS-CoV-2_α-gal_ will result in subsequent transcription and translation of the gene in infected host cells. This will further result in the production of many copies of the viral α1,3GT that will be incorporated into the trans-Golgi compartment of the infected cells. In that compartment, the viral α1,3GT will synthesize α-gal epitopes on many of the viral glycans. Such synthesis will result in the formation of SARS-CoV-2_α-gal_ virions with multiple α-gal epitopes on their glycan shield. It could be argued that propagating enveloped viruses in non-primate host cells containing active α1,3GT may result in similar formation of α-gal-presenting virions, as previously shown with various viruses [52,53,54,55,56,57,58,59,60,61]. However, due to the competition between α1,3GT and other glycosyltransferases of various potencies at the trans-Golgi compartment (e.g., sialyltransferases) [110], the number of α-gal epitopes per virus in some vaccines may be suboptimal for inducing the process illustrated in Figure 2A. Moreover, human and Old World monkey host cells for viral propagation lack α1,3GT. The translation of the viral α1,3GT at high concentration by the many copies of the arming *GGTA1* viral gene increases the probability for synthesis of multiple α-gal epitopes on the viral glycan shield.

Arming the SARS-CoV-2 virus with the *GGTA1* gene is feasible by standard genetic engineering methods, as suggested by studies in the following three viruses, which have been engineered to include the *GGTA1* gene in their genome for purposes other than an inactivated whole-virus vaccine.

*Adenovirus*—Adenovirus was the first virus that was armed with the *GGTA1* gene in its genome. For this purpose, the open reading frame of mouse α1,3GT cDNA was inserted into an adenovirus genome in which the early genes E1 and E3 were deleted [76,111]. Ad_α-gal_ was propagated in the human 293 kidney cell line, which contains the E1 complementary viral gene. Analysis of the viral α1,3GT activity following infection by Ad_α-gal_ of the human HeLa cancer cell line led to the detection of the *GGTA1* mRNA within 4 h post-transduction. Presentation of α-gal epitopes on the cell membrane was detected after 10 h, and it reached maximum presentation of ~2 × 10^6^ epitopes/cell within 24 h [111]. A similar viral *GGTA1* gene expression was observed following transduction of mouse B16 melanoma cells [76]. Irradiated B16_α-gal_ cells were found to be a much more effective vaccine that induces a protective immune response against a challenge of the vaccinated GT-KO mice with live B16 melanoma cells than vaccination with irradiated B16 cells lacking α-gal epitopes [76].

*Newcastle disease virus (NDV)*—NDV armed with the porcine *GGTA1* gene (NDV_α-gal_ also called NDV-GT) was studied as an oncolytic virus in cynomolgus monkeys and in humans with solid tumors [82]. The porcine *GGTA1* gene was inserted into the NDV genome using the NDV parental strain plasmid and helper plasmids [82]. Pre-clinical studies in cynomolgus monkeys carrying primary hepatocellular carcinoma have shown that intravenous administration of NDV_α-gal_ resulted in specific penetration of this virus into the hepatocarcinoma cells, followed by expression of α-gal epitopes on the infected tumor cells and binding of anti-Gal to these cells. This binding resulted in anti-Gal-mediated killing of tumor cells and conversion of the lesion into vaccines against the autologous hepatocarcinoma antigens. This resulted in the complete eradication of the tumor lesions within 3–4 months [82]. No eradication of the hepatocarcinoma lesions was achieved in cynomolgus monkeys treated with NDV lacking α-gal epitopes. A similar NDV_α-gal_ treatment of 20 cancer patients with solid tumors, at advanced stages of the disease, showed in 90% of the patients marked prolongation of survival of patients with stable disease and those with partial remission, as well as one patient displaying complete remission [82]. No serious adverse events were observed in the treated patients. These studies suggest that presentation of α-gal epitopes on NDV_α-gal_-infected tumor cells converts them into effective autologous vaccines of tumor antigens by a mechanism analogous to that in Figure 2A [82,83]. Furthermore, the successful conservation of *GGTA1* activity in the relatively small genome of NDV-GT (~16 Kb) suggests that insertion of this gene may be feasible in many different enveloped viruses used as inactivated whole-virus vaccines.

*Influenza virus*—Insertion of the *GGTA1* gene into influenza virus (NA_α-gal_ also called NA-GT) [112] was feasible by insertion of the mouse *GGTA1* gene into an influenza virus plasmid expressing NA vRNA [113]. The purpose of this influenza virus engineering was to generate an attenuated influenza virus vaccine that induces α-gal epitope expression on infected cells. Binding of the natural anti-Gal antibody to such infected cells is expected to result in their opsonization for phagocytosis by APCs of a mixture of viral antigens synthesized and cell membranes within these cells, thereby inducing an effective anti-influenza virus protective immune response [112]. The NA_α-gal_-attenuated virus was found to induce α-gal epitope expression on cells infected by it. Moreover, anti-Gal-producing GT-KO mice immunized with NA_α-gal_ were fully protected against this H1N1 virus and against lethal H5 or H3 virus challenges, whereas in the absence of anti-Gal, protection in immunized mice was only marginal. These findings imply that the increased immunogenicity of the attenuated NA_α-gal_ vaccine induces broadly cross-reactive, effective immune responses against conserved antigens in different influenza virus subtypes [112].

The studies of these three α-gal viral vaccines demonstrated the synthesis of α-gal epitopes in virus-infected cells, as a result of the viral α1,3GT activity. However, these studies did not determine the expression of α-gal epitopes on the infecting virions. It is probable, however, that NDV_α-gal_ and NA_α-gal_ present multiple α-gal epitopes. This assumption is supported by studies that demonstrated α-gal epitope presentation on envelope glycoproteins of viruses that replicate in mammalian cells containing α1,3GT and by studies demonstrating anti-Gal and complement-mediated neutralization of different viruses propagated in such mammalian cells [52,53,54,55,56,57,58,59,60,61]. Moreover, vesicular stomatitis virus and measles virus replicated in human cells transfected with the *GGTA1* gene also presented α-gal epitopes, bound serum anti-Gal, and were killed by it [55,61]. Thus, it is probable that enveloped viruses armed with the *GGTA1* gene will serve as effective inactivated α-gal whole-virus vaccines that elicit a potent protective immune response with long-term immune memory.

## 11. Suggested Arming Inactivated Whole-Virus Vaccine with the *GGTA1* Gene: Ebola Virus as an Example

The Ebola virus belongs to the Filovirus group, which also includes Marburg viruses and Cueva viruses. Filoviruses cause severe and often fatal hemorrhagic fever in humans. The clinical treatments for the prevention of filovirus infections have not been fully established, yet [114,115]. Since its discovery in central Africa in 1976, five species of Ebola virus have been isolated, of which at least three species have been associated with lethal outbreaks [116,117]. A few vaccines have been studied with mixed results in monkeys, including inactivated whole-virus vaccines, virus-like particles, viral vectors or plasmid DNA expressing filovirus genes, which were studied as vaccine platforms [114,115,118,119]. Recently, mRNA vaccines were reported to be successful in mice [120]. It is of note that the co-evolution of African monkeys with Filoviruses, for millions of years, could have resulted in their increased innate immune resistance to these viruses, which may not have occurred in humans. Thus, some innate immune protection to Filovirus infections may be in monkeys but not in humans.

It is suggested here that the inactivated Ebola virus (EBOV) vaccine engineered to present α-gal epitopes on N-linked glycans of the envelope glycoprotein (i.e., EBOV_α-gal_) may be beneficial in comparison to other vaccines, such as mRNA or DNA vaccines of the envelope glycoprotein (GP), for several reasons: 1. The *N*-linked glycosylation sites on Ebola virus envelope glycoprotein reveal a dense glycan shield on the virus that protects it against the human immune system [117,121,122]. There are 17 N-linked carbohydrate chains on each GP protein molecule and mucin-like regions that mask the antigenic peptides of this protein [20,21,122]. The presence of several SA units on each GP molecule may further decrease the uptake of such molecules produced by mRNA vaccines due to their repulsion from APCs by the ζ-potential. It is suggested here that effective anti-GP antibody production and a long-term immune memory for this antibody following inactivated EBOV_α-gal_ vaccination may enable the neutralization of infecting Ebola virus. This neutralization may be mediated by high-affinity anti-GP antibodies produced in vaccinated individuals despite the masking activity of the glycan shield on infecting virions. 2. In addition to the GP envelope protein, the Ebola virus contains several proteins that can serve as targets for the destruction of Ebola-infected cells by CTL. These proteins include nucleoprotein (NP), RNA-dependent RNA polymerase (L), matrix protein (VP40), polymerase cofactor (VP35), transcriptional activator (VP30), and secondary matrix protein (VP24) [114,115]. Some of the proteins (e.g., VP40) were found to rearrange their structure and fulfill more than one function [123]. It is suggested that a robust Ebola-specific T cell response against all these antigens within a multi-antigenic EBOV_α-gal_ inactivated whole-virus vaccine may result in the formation of many memory CTLs capable of killing cells infected with the virus at early stages of the infection, thus preventing its expansion. In addition, such an immune response may destroy potential Ebola virus variants with immune escape mutations in the GP protein. 3. Whereas the GP envelope protein may vary in the different Ebola species, the internal proteins may have conserved structures. Therefore, the EBOV_α-gal_-inactivated whole-virus vaccine of one species may elicit a CTL immune response that cross-reacts against several viral proteins in other species, as well. No information exists so far on engineering the Ebola virus to present α-gal epitopes on the GP protein. Expression of these epitopes on vaccinating virions may be achieved by arming them with the *GGTA1* gene, as described above for SARS-CoV-2.

## 12. Limitations

### 12.1. Yield of Virus Containing the GGTA1 Gene

Production of the designed live attenuated influenza virus NA_α-gal_ in Madin–Darby canine kidney (MDCK) cells was found to be lower by 2.5 log in virus titer than the wild-type influenza virus [112]. The reason for this decrease in yield is not clear and may be associated with the location of the inserted *GGTA1* gene in the viral genome, stability of the virus with an altered glycan shield, or other unknown reasons. Significant decrease in the yield of vaccinating α-gal virions may require insertion of the *GGTA1* gene in other parts of the genome, evaluation of the yield, and comparative studies of the yield in several host cell lines. If the yield remains suboptimal following such attempts, alternative options for production of viruses presenting α-gal epitopes may be considered [100], including the following: 1. in vitro synthesis of α-gal epitopes on the glycan shield of the inactivated virus using recombinant α1,3GT as in the enzymatic reaction described above. If the glycan shield of the virus presents terminal sialic acid, as in Figure 1A, neuraminidase, which cleaves the sialic acid, is added to the enzymatic reaction [13]; 2. use of cell lines having engineered to have several copies of the *GGTA1* gene; 3. transduction of host cells with replication-defective Ad_α-gal_ virus prior to host cell infection with the virus propagated for vaccine preparation [111].

### 12.2. Limitation in Use of Attenuated Virus Vaccines

The synthesis of α-gal epitopes on the glycan shield of attenuated virions used as vaccines may add an element of uncertainty to the efficacy of such vaccines. Viruses presenting α-gal epitopes bind anti-Gal, and when incubated in human serum, they are killed by the activated complement system [53,54,55,56,57,58,59,60,61]. Cells infected by the virus containing the *GGTA1* gene are further induced to present α-gal epitopes, bind anti-Gal, and are killed both by complement activation and by antibody-dependent cell cytolysis (ADCC) [82,112]. The resulting uptake of the killed viruses and killed infected cells by APCs increases the effectiveness of viral antigens as immunizing vaccines [112]. However, these anti-Gal-mediated killing mechanisms may also prevent the expansion of the attenuated virus, particularly in individuals with high titers of this antibody. Therefore, the efficacy of attenuated α-gal virus vaccines may vary from one individual to another, since in some individuals the virus may disappear shortly after its administration into the treated individual. The use of inactivated α-gal whole-virus vaccines ensures their robust targeting to APCs and the effective immune response (as in Figure 2A) at both high and low titers of the natural anti-Gal antibody [28].

### 12.3. Limitation Due to the α-Gal Syndrome

A general limitation for the use of various α-gal therapies in humans is in the treatment of individuals with the α-gal syndrome. These individuals produce anti-Gal IgE following bites by the tick *Ambliyomma americanum* (the “lone star” tick) in the USA [124,125,126,127], *Ixodes ricinus* in Europe [128], *Haemaphysalis longicornis* in Asia [129], and *Ixodes holocyclus* in Australia [130]. Because of the produced anti-Gal IgE, individuals with the α-gal syndrome are allergic to α-gal epitopes in meat (beef, pork and lamb). α-Gal epitopes on glycolipids, glycoproteins and proteoglycans released by meat digestion bind to anti-Gal IgE on mast cells and basophils [124,125,126,127,128,129,130,131]. The resulting degranulation of these cells induces, within several hours, a variety of allergic reactions such as rash, hives, nausea or vomiting, difficulty in breathing, low blood pressure, diarrhea, stomach cramps, and pain. Thus, recipients of α-gal vaccines should be asked prior to vaccination if they have the α-gal syndrome and whether they are allergic to meat. Such individuals should receive allergy-suppressing medication prior to vaccination and one or a few days post vaccination with α-gal virus vaccines.

## 13. Concluding Remarks

Production of the natural anti-Gal antibody in large amounts in all humans who are not severely immunocompromised provides a novel approach for increasing the effectiveness of vaccines that protect against infection by enveloped viruses. The ligand of anti-Gal is the α-gal epitope, which is a carbohydrate antigen with the structure Galα1-3Galβ1-4GlcNAc-R. Inactivated whole-virus vaccines engineered to present α-gal epitopes on their glycan shield (α-gal virus vaccines) form immune complexes with anti-Gal following their injection into vaccinated individuals. The anti-Gal/α-gal virus vaccine interaction activates the complement system, which induces extensive chemotactic recruitment of APCs to the vaccination site. Opsonization of the vaccinating inactivated α-gal virions by anti-Gal targets them for robust uptake by the recruited APCs. This mechanism results in transport by APCs of many more vaccinating virions to regional lymph nodes and processing of many more viral antigens than vaccination with virions lacking α-gal epitopes. Thus, the efficacy of α-gal virus vaccines is much higher than that of traditional inactivated whole-virus vaccines lacking α-gal epitopes. The α-gal whole-virus vaccines further induce a potent protective immune response and long-term immune memory against several viral antigens. Therefore, it is postulated that in individuals vaccinated with SARS-CoV-2_α-gal_ inactivated whole-virus vaccine, the potent immune response to M, E and N antigens will also protect against variants with mutations in the S-protein gene, which enable escape from anti-S-protein antibodies. A similar effective immune protection may be feasible with inactivated α-gal Ebola virus vaccines. The presentation of α-gal epitopes on α-gal viral vaccines can be achieved by arming enveloped viruses with the *GGTA1* gene. This gene encodes the α1,3galactosyltransferase enzyme that synthesizes α-gal epitopes on N-linked carbohydrate chains of the complex type on the glycan shield of enveloped viruses propagated in fowl or mammalian cells. Thus, engineering inactivated whole-virus vaccines to present α-gal epitopes on their glycan shield converts this shield from a portion of the virus that decreases its immunogenicity into a carbohydrate layer of the virus that markedly increases its immunogenicity by recruiting APCs to the vaccination site. This engineering furter targets the multi-antigenic vaccinating virions for robust uptake by APCs, which transport large amounts of processed vaccinating antigens to draining lymph nodes. The ultimate result of this process is a marked increase in effectiveness of the α-gal virus vaccines in comparison to the same virus vaccines lacking α-gal epitopes.

## Data Availability

The data in this review are available upon request.

## Abbreviations

The following abbreviations are used in this manuscript:

α-gal	carbohydrate antigen with the structure Galα1-3Galβ1-4GlcNAc-R
α1,3GT	α1,3galactosyltransferase
ADCC	antibody-dependent cell cytolysis
Ad_α-gal_	adenovirus containing the *GGTA1* gene
APCs	antigen-presenting-cells
CR1	complement receptor 1
CTL	cytotoxic T cells
FcγR	Fcγ receptor
GT-KO	mouse knockouted for the α1,3galatosyltransferase gene (*GTTA1*)
HLA	human leukocyte antigen
NDV	Newcastle disease virus
NDV-GT	Newcastle disease virus armed with the *GTTA1* gene
TCR	T cell receptor
